# The relationship between the abundance of the Nigeria-Cameroon chimpanzee (*Pan troglodytes ellioti*) and its habitat: a conservation concern in Mbam-Djerem National Park, Cameroon

**DOI:** 10.1186/s12898-018-0199-3

**Published:** 2018-10-01

**Authors:** Serge Alexis Kamgang, Kadiri Serge Bobo, Fiona Maisels, Ruffin Dupleix Delarue Ambahe, Désiré Edgar Ambassa Ongono, Mary Katherine Gonder, Paul Johnson, Jorgelina Marino, Brice Sinsin

**Affiliations:** 1 Garoua Wildlife School, Face aéroport international de Garoua, P.O. Box 271, Garoua, Cameroon; 2 Department of Forestry, Faculty of Agronomy and Agricultural Sciences, University of Dschang, P.O. Box 222, Dschang, Cameroon; 3 Global Conservation Program, Wildlife Conservation Society, 2300 Southern Boulevard, Bronx, New York, NY 10460 USA; 4 Faculty of Natural Sciences, University of Stirling, Stirling, FK9 4LA Scotland UK; 5 Cameroon Biodiversity Programme, Wildlife Conservation Society, P.O. Box 3035, Yaoundé, Cameroon; 6 Ministry of Forestry and Wildlife, Yaounde, Cameroon; 7 Department of Biology, Drexel University, Philadelphia, PA 19104 USA; 8 Wildlife Conservation Research Unit, Department of Zoology, University of Oxford, Recanati-Kaplan Centre, Tubney, Oxford UK; 9 Laboratory of Applied Ecology, Faculty of Agronomic Sciences, University of Abomey-Calavi, 01 P.O.Box 526, Cotonou, Benin

**Keywords:** Great apes, Mbam-Djerem National Park, *Pan troglodytes ellioti*, Habitat variation, Nest abundance, Distance sampling

## Abstract

**Background:**

Understanding the relationship between great apes and their habitat is essential for the development of successful conservation strategies. The chimpanzee *Pan troglodytes ellioti* is endemic to Nigeria and Cameroon, and occupies an ecologically diverse range of habitats from forests to forest-savannah mosaic in Mbam-Djerem National Park (MDNP) in Cameroon. The habitat variation in chimpanzees is poorly understood in MDNP which provides an excellent opportunity to assess ecological factors that shape the abundance and distribution patterns of *P. t. ellioti* over a small geographic scale.

**Results:**

We counted 249 nests along 132 km of transects in total. Of these, 119 nests along 68 km occurred in dense forest and 130 nests along 64 km in forest-savannah mosaic. Chimpanzee density was 0.88 [95% CI (0.55–1.41)] individuals/km^2^ in the dense forest and 0.59 [95% CI (0.19–1.76)] in the forest-savannah mosaic. Nest abundance varied with vegetation type and was higher in areas with dense canopy cover, steeper slopes and relatively higher altitudes.

**Conclusions:**

Our estimates of chimpanzee densities were lower than reported in other studied populations in the range of the Nigeria-Cameroon chimpanzee. However, we found that habitat features, slope and altitude likely play a role in shaping patterns of chimpanzee nesting ecology. Further studies need to be focused on nest decay rates and phenology of useful plants in order to model chimpanzee abundance and distribution in Mbam-Djerem National Park.

**Electronic supplementary material:**

The online version of this article (10.1186/s12898-018-0199-3) contains supplementary material, which is available to authorized users.

## Background

Great ape populations are currently threatened by hunting, habitat loss and infectious diseases [1, 2]. Understanding the relationship between each great ape species and its environment is therefore crucial for developing conservation policy [3]. For chimpanzees, key requirements such as food and nesting materials are sensitive to environmental variation, including climate change and other anthropogenic factors such as habitat conversion and poaching. However, monitoring chimpanzee population size is inherently difficult, and few studies have demonstrated clear links between habitat variation and conservation value [4, 5]. The Mbam-Djerem National Park (MDNP) in Cameroon offers an excellent opportunity to assess ecological factors shaping the abundance and distribution of the Nigeria-Cameroon chimpanzee (*Pan troglodytes ellioti*) over a small geographic extent in the core zone of the protected area, which includes both dense forest, colonizing forest and savannah ecosystems. Until now, the distribution pattern and abundance of the Nigeria-Cameroon chimpanzee has not been completely understood in MDNP which may hamper their long-term conservation. Our research highlights this issue by providing data on chimpanzee density and the environmental drivers affecting their distribution. Moreover, as the forest is currently expanding [6] and replacing savannah in MDNP [7], understanding how chimpanzees use different habitats can inform conservation efforts by providing key monitoring parameters on behalf of this species.

Studies of the subspecies *P. t. ellioti* in the dry and gallery forests of Nigeria in Gashaka-Gumti National Park [8], in Cameroon at Ebo Forest [9] and MDNP have so far failed to address the relationship between abundance and habitat characteristics. Differences between chimpanzee populations regarding ecology, social organisation and genetics [10, 11], population size [12], home range size [13], feeding habits [14, 15] and nesting behavior [16] have been described, and appear to be related to differences in habitat types [17, 18], but few studies have quantified how these factors impact local population sizes and habitat use [19]. Habitat assessment between Mahale Mountains and Gombe in Tanzania [20], at Lagoas de Cufada National Park in Guinea-Bissau [18], and between forests of Western Uganda [21] and Mount Assirik in Senegal [13, 14, 22] are examples of studies comparing chimpanzee ecological behavior across habitat types. Other studies have explored chimpanzee diet and habitat selection in the Democratic Republic of Congo [23] and in Uganda [15], and nesting ecology in Nigeria [24, 25] and Tanzania [16]. Little is known, however about the Nigeria-Cameroon chimpanzee in MDNP.

The Nigeria-Cameroon chimpanzee was recognized since 1997 as the fourth subspecies of chimpanzees [26–28] and is the least studied among all subspecies of chimpanzees. Classified as Endangered by IUCN [29], with between 3500 and 9000 individuals remaining [25, 28, 29], their populations size is declining across their limited natural range [29]. As is the case for other subspecies of chimpanzees, landscape fragmentation, habitat loss, disease, commercial bushmeat hunting and climate change are all substantial threats to the conservation of the Nigeria-Cameroon chimpanzee [29]. The conservation status of this subspecies may also change rapidly in response to habitat change [29]. It is therefore important to explore how habitat variation impacts the density and distribution of local chimpanzee populations.

Emerging methods such as the use of infra-red camera [30], the use of drones [31] and genetic material are also appropriate to reliably estimate the density and distribution of chimpanzee communities [32], but these studies are currently limited to relatively small areas and are outside the budgetary capacities of most protected area management plans within the country. Studying the distribution of nests is currently the most efficient means to estimate the distribution and density of chimpanzee populations [12, 33]. Although evidence of presence such as direct sighting, feeding remains and footprints are still frequently used to derive densities of chimpanzees, the most robust method of estimating population density continues to be based on nest counts [34]. The main objective of this study was to estimate the density of the Nigeria-Cameroon chimpanzee in two main habitat types within the MNDP, namely forest-savannah mosaic and dense forest, and to study the nesting ecology of chimpanzees in these two main habitat types. We investigated how habitat variation in the forest-savannah mosaic and in the dense forests affects chimpanzee distribution in MNDP, and the importance of the availability of nesting materials, canopy cover, understory, slope and altitude. The results will help to design regular monitoring activities focusing on chimpanzee habitat suitability and to shape effective management practices in MDNP. Key activities might be focused on the phenology of useful plants for chimpanzees as well as human encroachment in their suitable habitats. Furthermore, the findings will be relevant to the update of the imminent revision of the 2011 IUCN Regional Conservation Action Plan for the subspecies [28].

## Methods

### Mbam and Djerem National Park

**Fig. 1:**
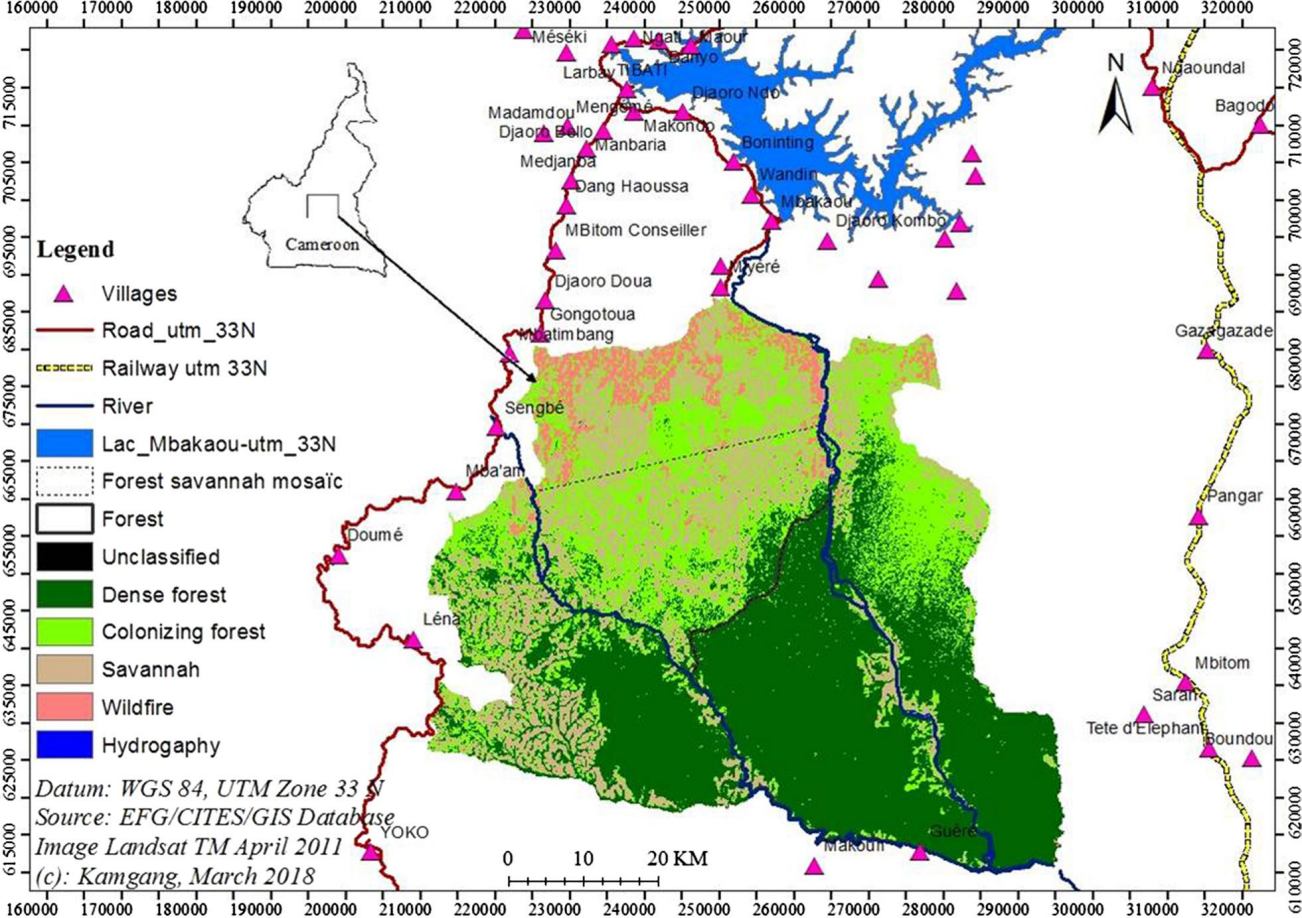
The study area with the 4165.2 km^2^ Mbam-Djerem National Park (MDNP). The 1662.34 km^2^ core zone in the middle of MDNP is delimited with rivers. The Mbakaou artificial lake in the northern periphery of the park is shown in blue. The inset represents the location of MDNP within Cameroon

Created in 2000, MDNP covers 4165.2 km^2^ and lies between 5°30′N and 6°14′N, and 12°20′E and 13°15′E [35] (Fig. 1). The rainy season extends between mid-April and mid-October and a dry season between mid-October and mid-April. Average rainfall is 1900 mm/year, average annual temperature is 24 °C [35]. The area lies within the Guinea-Congolia/Sudania regional transition zone, between the Soudanian regional centre of endemism in the north and the Guinea-Congolian forest block in the south [36]. The vegetation of the MDNP grades from savannah in the northwest through forest-savannah mosaic to closed canopy humid forest in the south-west [7] (Fig. 1). The relief is relatively flat and the altitude ranges from 650 to 930 m above sea level (a.s.l.). Approximately 30,000 human inhabitants live in 74 villages at the periphery of the MDNP [35]. These people mostly depend on natural resources for their food, traditional medicine and income. The human population tends to be concentrated in the northern periphery where grazing lands are available and where the Mbakaou Dam was constructed in 1964, and in the eastern periphery of the MDNP where the Belabo-Ngaoudéré railway link is found as established in 1970 [35] (Fig. 1).

### Survey design

We used data from a transect survey for large mammals collected between 2009 and 2014 to design a sampling plan for the assessment of chimpanzee density at MDNP. We used the encounter rate of chimpanzee nests derived from these previous surveys as an indicator of the effort required to obtain a density estimate. The coefficient of variation of 15% for forest-savannah mosaic and 20% for dense forest were chosen to perform equation 7.3 [37].

**Fig. 2:**
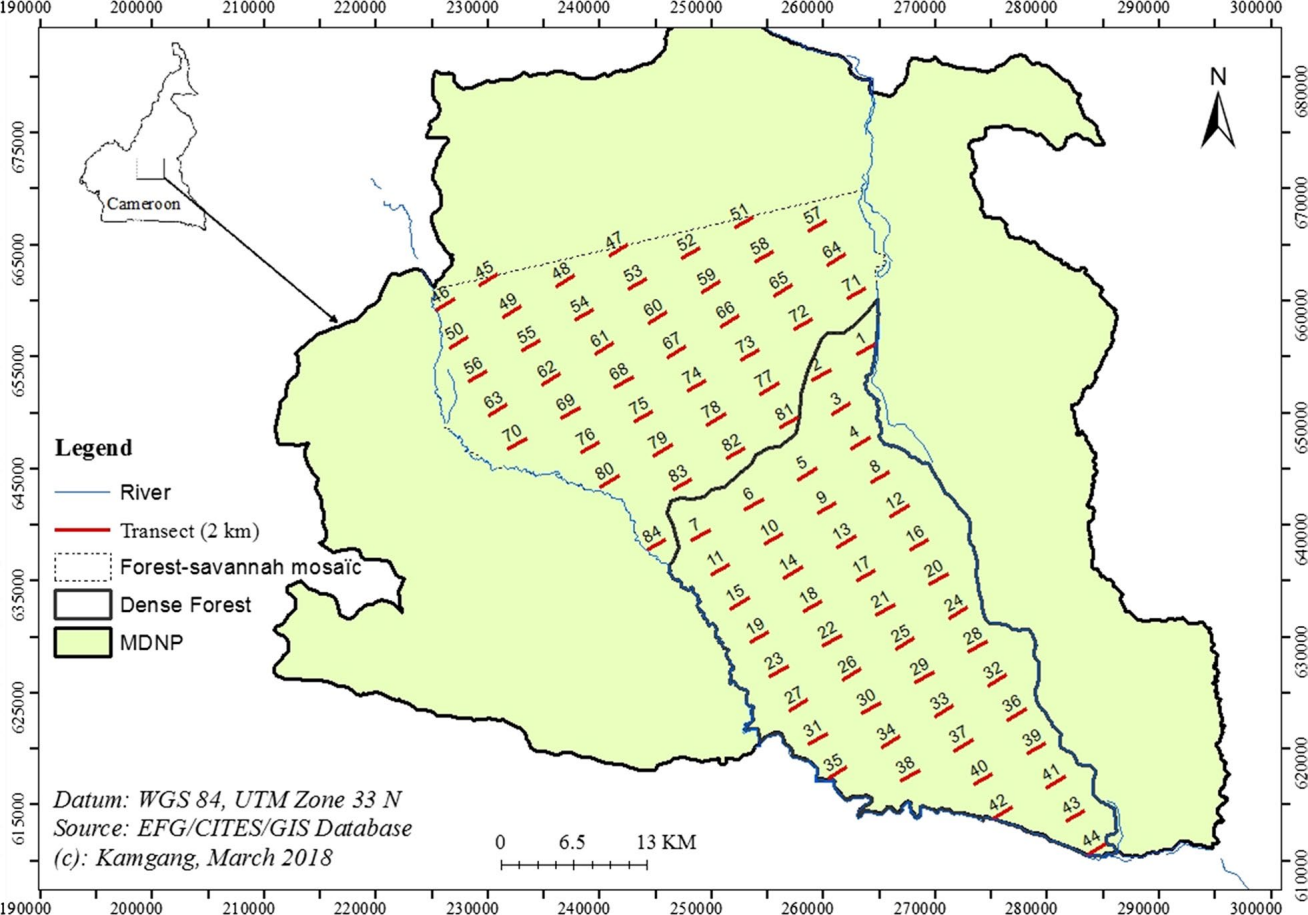
Survey design of the assessment of chimpanzee abundance within the core zone of MDNP

Thus, given the encounter rates of 1.7 nest/km and 0.93 nest/km, we found that 80 km and 88 km of effort, respectively in forest-savannah mosaic and in dense forest, were required to assess the chimpanzee nest density with the defined target precision. The effort to reach the specific target coefficient of variation was calculated using the value of 3 as dispersion parameter (b) in equation 7.3. Using these results, we developed a population survey protocol that included 84 transects of 2 km each (Fig. 2). The core zones included two strata (based on the physiognomy and structural characteristics using satellite imagery Landsat TM of April 2011) within the forest-savannah mosaic in the north and the dense forest in the south.

### Standing crop nest count

Chimpanzees are elusive, difficult to see and occur at relatively low densities [28, 38–40], thus requiring an indirect method for density estimation. Nest counts are often used as all weaned chimpanzees from around 3 years of age generally build a night nest to sleep in [41]. We used the standing crop nest count (SCNC) method [42, 43] to estimate the density of chimpanzees by completing unrepeated transects to count nests from January to June 2016 at MDNP. Two survey teams of six persons each comprising one MDNP biologist team leader, two rangers and three local guides were established. The teams were trained on the use of CyberTracker to collect field data following the Wildlife Conservation Society protocol [44]. Along each transect, the coordinates of each nest were recorded as well as the age class of each nest. The age class of each nest was classified using the system developed by Tutin and Fernandez [41] as *fresh* (vegetation is still green, leaves are not yet wilted and urine and faeces may be present at the site); *recent* (nest contains leaves that are green but wilted); *old* (nest has leaves that are no longer green but remain intact); and *rotting* (nest has shed its leaves, leaving only bare branches).

### Habitat assessment

Predicting the influence of habitat attributes on wildlife is useful for conservation and protected area management [45–48]. While walking along transects, we examined a set of variables to assess the habitat used by chimpanzees. For each nest encountered, we recorded the distance along the transect with a topofil, the perpendicular distance to the transect line of each nest spotted from the transect (using tape measure), the type of nest, the height above ground, the tree height, the tree species, and the diameter at breast height (dbh) of the tree (i.e. at 1.3 m above ground), the slope of the site, the vegetation type, the canopy cover and the age class of each nest in the nesting site. Slope was defined according to the following scheme: 0 = flat, 1 = low, 2 = moderate and 3= steep. Nest type was defined according to [49] as (a) Minimum (terrestrial nest with one or two stems of herbaceous plants); (b) Mixed (terrestrial nest with herbaceous plants and woody vegetation); (c) Tree (nest made in tree). The canopy cover was assigned as open (0–25%), low closure (26–50%), moderate closure (51–75%) and high closure (> 75%).

Habitat type included seven categories [50]: (a) Colonising Forest (CF); (b) Gallery Forest (GF); (c) Liana Forest (LF); (d) *Marantaceae* Forest (MF); (e) Mixed Forest with Closed Understory and *Marantaceae* (MFCUM); (f) Mixed Forest with Closed Understory (MFCU); (g) Mixed Forest with Opened Understory (MFOU). These habitats are described in detail in Additional file 1. The plant species used for nesting were identified in the field. We used the Garoua Wildlife School herbarium to identify nesting plants from field samples. We assessed the relationship between habitat type and nest density using several parameters, including number of nests, nest height, dbh of the nesting tree, and encounter rates of nest sites registered for each strata and habitat type. We conducted non-parametric Kruskal–Wallis tests to compare dependent variables across habitat types and strata. We also used General Linear Models and contingency table [51] to explore the effects of habitat attributes (e.g. plant species, dbh of the nesting tree, slope, understory, canopy cover and altitude) on the nesting sites (e.g. nest abundance, nest height, nest encounter rate). We used the software program R for these analyses [51].

### Estimating chimpanzee density, population size and distribution

#### Conversion parameters

Converting nest density into an estimate of chimpanzee density requires two parameters: a nest production rate and a nest decay rate [41, 42, 52]. Nest decay data were not available for MDNP. Therefore, similar to other studies where reliable nest production and decay data were unavailable, we used a range of possible nest decay rate values from previous studies [33, 52] to estimate the density of chimpanzees for this study. Nest production rates are estimated by averaging the number of nests built per day by a weaned chimpanzee from direct monitoring of habituated chimpanzees to then assess potential nest production rates in the given study site [42, 53]. Weaned chimpanzees generally make new sleeping nests every night [54] but they also sometimes build day nests in which to rest. Allowing for this, we used a value of 1.09 (± 0.5) nests per day reported in previous research [39, 42] for the present study.

Measuring nest decay rates is more challenging, as it involves monitoring a sufficient number of fresh nests from the time they are built to the time they disappear [39]. In addition, nest decay rates may vary considerably depending on the plant species used to build the nest and the local climatic parameters and therefore vary considerably between sites and vegetation type [33]; thus carrying an associated error which may affect the precision of density estimates [33, 53, 55]. Moreover, climate can affect the rate of re-use and building of nests as well as decay rates [21, 39, 54, 56]. Observations in Ebo Forest, Cameroon, which is close to the MDNP, suggest a nest decay rate of 88.2 (± 7.1) days. This is similar to the estimate from the Taï forests in the Ivory Coast (91.22 ± 5.8 days) [12]. Both MDNP and Ebo forest are found in the northern side of the Sanaga River and within the same climatic domain of Cameroon (2°–6° of northern latitude), with a similar amount of annual rainfall (2400 mm in the Ebo Forest and 1900 mm in the MDNP). Much uncertainty remains, however, in these estimates [43, 57]. A value of 221 days was previously used in the MDNP to convert nest density into chimpanzee density. To assess the sensitivity of the density estimate to nest decay rate, we also used 120 and 221 days.

#### Conversion of nest density to chimpanzee density

We used the Distance 7.0 Program to derive nest and chimpanzee density estimates [37, 58]. This program implements a series of detection function models with their expansion series acquired from the data set (Additional file 2) to estimate the chimpanzee density by inference from the nest density [37]. Different models are then compared based on the Akaike Information Criterion [58].

Single nests have been used to estimate chimpanzee nest density [12, 59]. Chimpanzee density was then derived from nest density using conversion parameters (nest production rate, nest decay rate and the proportion of nest builders).

#### Chimpanzee distribution

We calculated encounter rates of nest sites for each transect and used this information to develop Inverse Distance Weighting-IDW interpolation using 30 neighbors and a power of 2 in ArcGIS 10.1 software [60, 61]. The corresponding raster layer was extracted by mask and exported as PNG file.

## Results

### Detection models

**Fig. 3:**
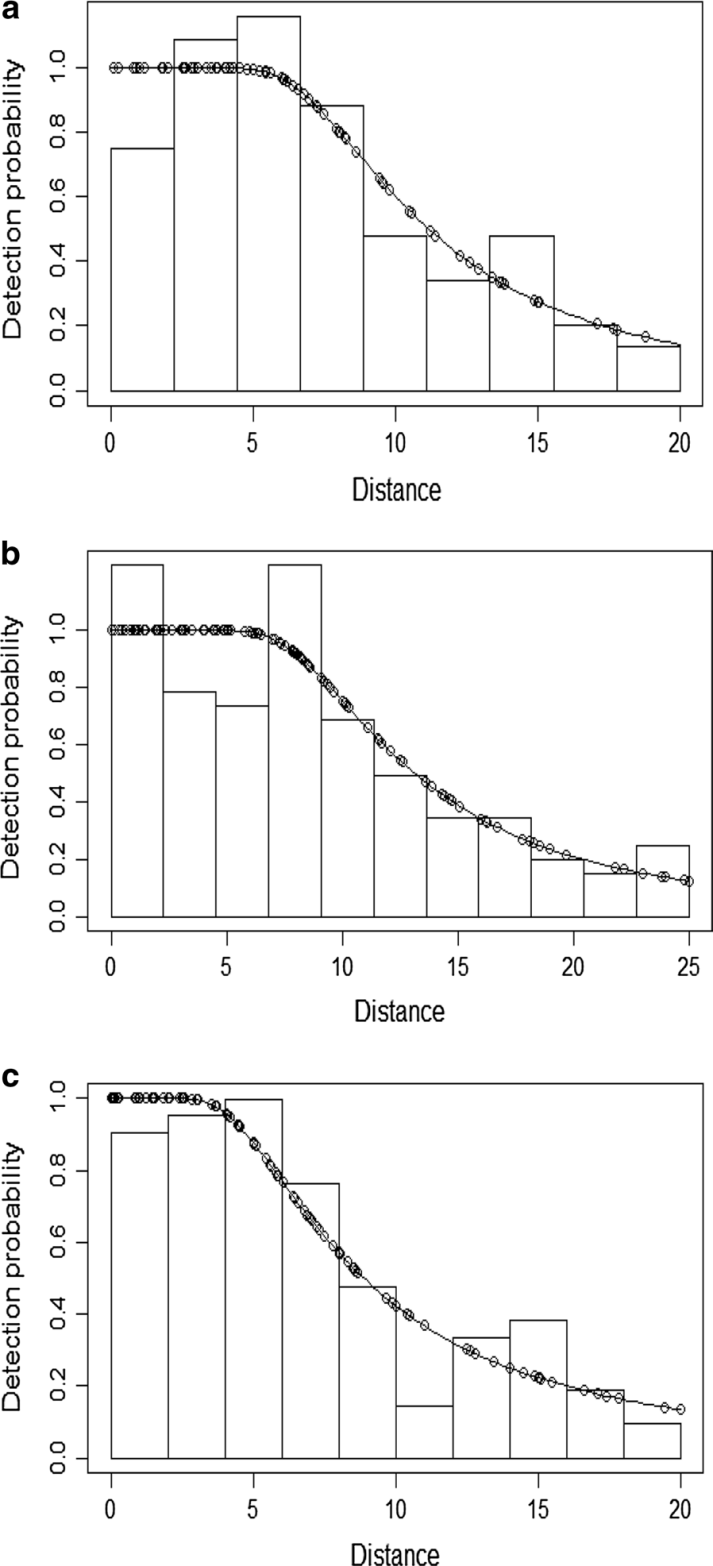
**a** Global detection function curve of all nests combined (Hazard-rate simple polynomial) truncated at 20 m of perpendicular distance. **b** Global detection function curve of nests from the forest-savannah mosaic (Hazard-rate simple polynomial) truncated at 25 m of perpendicular distance. **c** Global detection function curve of nests from the dense forest (Hazard-rate cosine) truncated at 20 m of perpendicular distance

The model fits for the nest counts were as follows: hazard-rate simple polynomial truncated at 20 m of perpendicular distane for all data (Fig. 3a), Hazard-rate simple polynomial truncated at 25 m in the forest-savannah mosaic (Fig. 3b) and Hazard-rate cosine truncated at 20 m in the dense forest (Fig. 3c).

### Chimpanzee density at Mbam-Djerem National Park

A total of 32 transects were surveyed in the forest-savannah mosaic and 34 transects in the dense forest. We observed 249 nests from these transects, of which 119 nests occurred in dense forest and 130 nests in forest-savannah mosaic. The nest detection probability was 0.58 (± 0.05) and 0.52 (± 0.06), respectively, in the forest-savannah mosaic and the dense forest. The effective strip width was 14.62 (± 1.37) m in the forest-savannah mosaic and 10.46 (± 1.30) m in the dense forest. Table 1 shows nest density estimates while Table 2 shows chimpanzee density estimates.

**Table 1:** Estimated nest density (per km^2^) in the forest-savannah mosaic, the dense forest and all nests combined

	Number of Nests^a^	ESW (SD)	Pa (SD)	Nest density	CV	Models
All data	249	12.95 (1.07)	0.51 (0.04)	80.75 (51.32–127.04)	23.06	Hazard-rate simple polynomial + 20 m truncation
Forest-savannah mosaic	130	14.62 (1.37)	0.58 (0.05)	77.20 (36.06–165.29)	38.69	Hazard-rate simple polynomial + 25 m truncation
Dense forest	119	10.46 (1.30)	0.52 (0.06)	84.80 (52.97–135.74)	23.80	Hazard-rate cosine + 20 m truncation

*ESW* effective strip width (m), *SD* standard deviation; *Pa* probability of detection; *CI* 95% confidence interval, *CV* percentage coefficient of variation

^a^Sample size after truncation

**Table 2:** Estimated chimpanzee density and population size based on a range of estimated nest decay rate

Decay rate	88 days		120 days		221 days		Area (km^2^)
Estimates	Chimpanzees/km^2^ (CI)	Population size (CI)	Chimpanzees/km^2^ (CI)	Population size (CI)	Chimpanzees/km^2^ (CI)	Population size	
All data	0.83 (0.32–2.11)	1396 (535–3643)	0.61 (0.23–1.59)	1026 (397–2650)	*0.33* *(0.12–0.86)*	557 (216–1439)	1662.34
Forest-savannah mosaic	0.80 (0.26–2.41)	612 (203–1842)	*0.59* *(0.19–1.76)*	449 (150–1343)	0.32 (0.10–0.95)	244 (82–729)	900.84
Dense forest	*0.88* *(0.55–1.41)*	795 (496–1272)	0.64 (0.24–1.68)	584 (225–1517)	0.35 (0.13–0.91)	317 (122–824)	761.5

*CI* 95% confidence interval. Estimates derived from decays rate considered to be the most suitable for each habitat type are given in italics

The density varies considerably depending on the nest decay rate used. The nest decay rate is inversely proportional to the nest density. Considering the same nest decay rate, chimpanzee densities were similar across strata although with different confidence interval.

### Habitat assessment

Surveyed effort was assessed as well as the proportion of nests in each habitat type (Table 3).

**Table 3:** Total effort and proportion of nests per habitat type

Vegetation types	Effort (km)	Percent	Proportion of nests
CF	48	36.36	40.56
GF	46	34.85	37.75
LF	24	18.18	14.05
MFOU	6	4.55	4.41
MFCU	4	3.03	1.60
MF	2	1.52	0.80
MFCUM	2	1.52	0.80
Total	132	100.00	100.00

*CF* colonising forest, *GF* gallery forest, *LF* liana forest, *MF Marantaceae* forest; *MFCUM* mixed forest with closed understory and *Marantaceae*, *MFCU* mixed forest with closed understory, *MFOU* mixed forest with opened understory

**Fig. 4:**
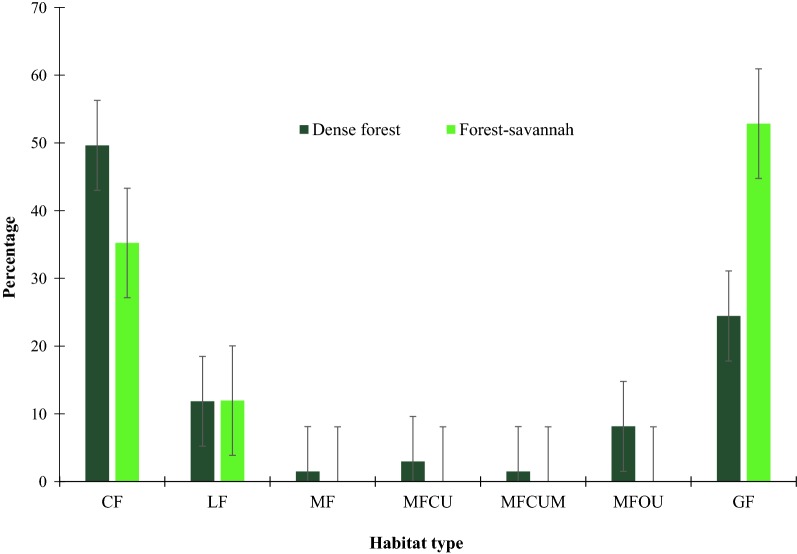
The percentage of nests per vegetation type in dense forest and in forest-savannah mosaic: *CF* colonising forest, *GF* gallery forest, *LF* liana forest, *MF Marantaceae* forest; *MFCUM* mixed forest with closed understory and *Marantaceae*, *MFCU* mixed forest with closed understory, *MFOU* mixed forest with opened understory

A total distance of 48 km (36.36%) was covered in colonising forest and 46 km (34.85%) in gallery forest. These are the two main vegetation types where chimpanzee nests were recorded. The proportion of nests varies with habitat types (F_6, 284_ = 9.54, P < 0.001). The number of nests found in colonizing forest was 101 nests (40.56%) and 94 nests (37.75%) in gallery forest (Fig. 4).

**Fig. 5:**
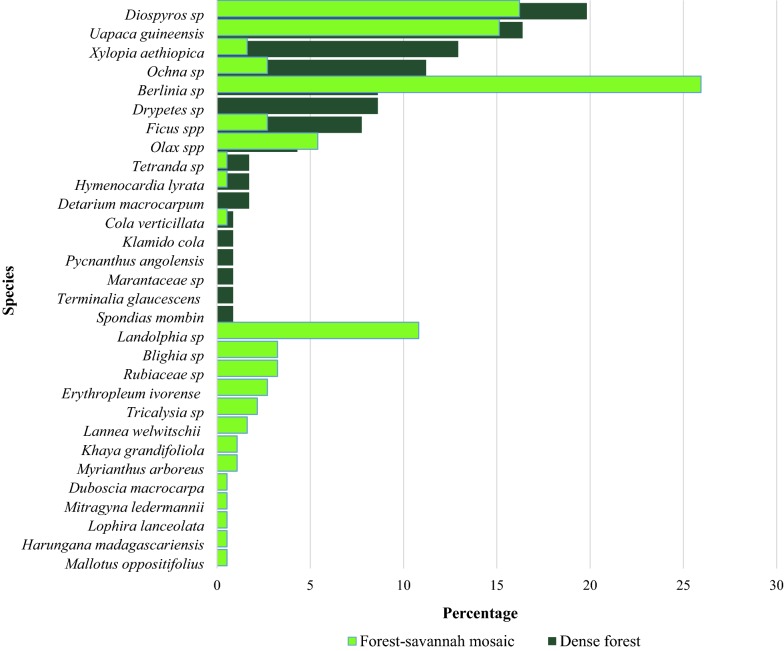
Nesting choice in different habitat types

We identified a total of 31 plant species used as nesting material. Species commonly used to build nests were *Berlina* sp. (*Caesalpiniaceae*) (18.84%), *Diospyros* sp. (*Ebenaceae*) (15.36%) and *Uapaca guineensis* (*Euphorbiaceae*) (14.78%) (Additional file 3) (Fig. 5).

No evidence for difference was found on the number of plant species used for nesting between the dense forest and the forest-savannah mosaic (Kruskal–Wallis X^2^ = 1.1, df = 1, P = 0.293) or between habitat types (Kruskal–Wallis X^2^ = 6, df = 6, P = 0.423).

There was no evidence for difference in nest density and chimpanzee density between the dense forest and the forest-savannah mosaic (Kruskal–Wallis X^2^ = 1, df = 1, P = 0.31). Similarly, there was no difference in nest encounter rates (Kruskal–Wallis X^2^ = 0.13, df = 1, P = 0.71) between the dense forest and forest-savannah mosaic.

The nest type frequency differed between dense forest and forest-savannah mosaic (Pearson’s X^2^ = 9.19, P = 0.046) and between habitat types (Pearson’s X^2^ = 14.84, P = 0.05). However, 98.88% of all nests (N = 249) were tree nests with 52.20% (N = 130) and 46.58% (N = 116) found, respectively in forest-savannah mosaic and in dense forest. Among all the nests registered (n = 249), only three (1.20%) were ground nests although there was no evidence as to whether chimpanzees slept in them over night or used them to rest during day. These nests were found in the dense forest and built with *Marantaceae* leaves (Additional file 4) (Table 4).

**Table 4:** Nest types per sector with the mean nest height and the mean dbh of the nesting tree

Sector	Area (km^2^)	Tree nests	Ground nests	Mean nest height (m)	Mean dbh of the nesting tree
Overall dataset	1662.34	246	3	11.59 ± 7.83	10.3 ± 8.42
Dense forest	900.84	116	3	10.61 ± 6.05	9.84 ± 5.72
Forest-savannah mosaic	761.5	130	0	12.42 ± 6.56	10.68 ± 6.10

Nest encounter rates varied significantly with altitude (F_1, 197_ = 55.24, P < 0.001). A total of 189 nests (75.9%) was observed between 650 m and 800 m a.s.l

There was a strong evidence of the influence of canopy cover (F_3, 284_ = 31.75, P < 0.001) and slope (F_3, 284_ = 10.22, P < 0.001) on the nesting site. In forest-savannah mosaic, 38.99% of the nests where found under high closure canopy (> 75%) while in dense forest, 37.03 of the nests were found under moderate closure canopy (51–75%). As for the slope, 53.45% of the nests recorded in forest-savannah mosaic and 60% in dense forest were found in low slope, although 31.44% of the nests were found in steep slope in forest-savannah mosaic. Low slope may offer comfortable nesting conditions to chimpanzee in MDNP.

**Fig. 6:**
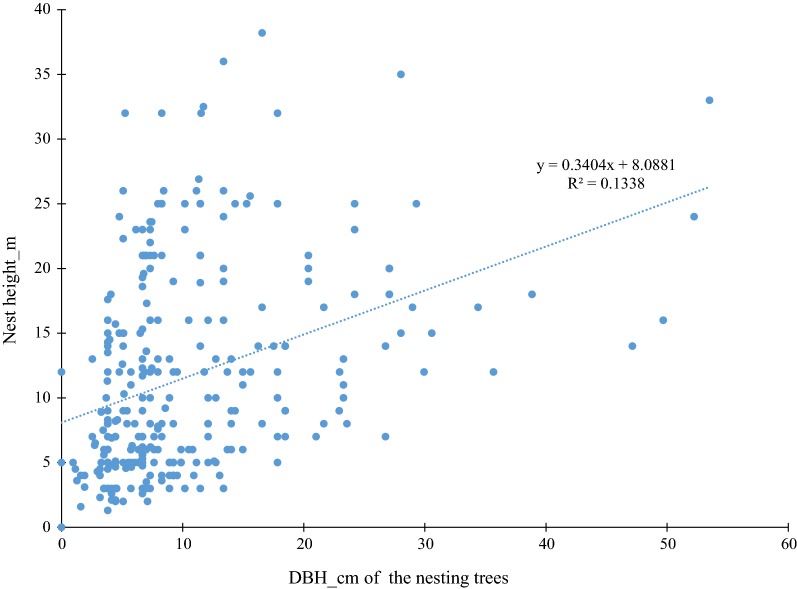
Correlation between the nest height and the diameter at breast height (dbh) of the nesting trees

There was a significant positive correlation between nest height and dbh of the nesting trees (Additional file 5) (r = 0.365, t_292_ = 6.717, p < 0.001) (Fig. 6). The mean dbh of nesting tree was 10.03 (± 8.42) cm, while the mean nest height was 11.59 (± 7.83) m.

### Chimpanzee distribution

**Fig. 7:**
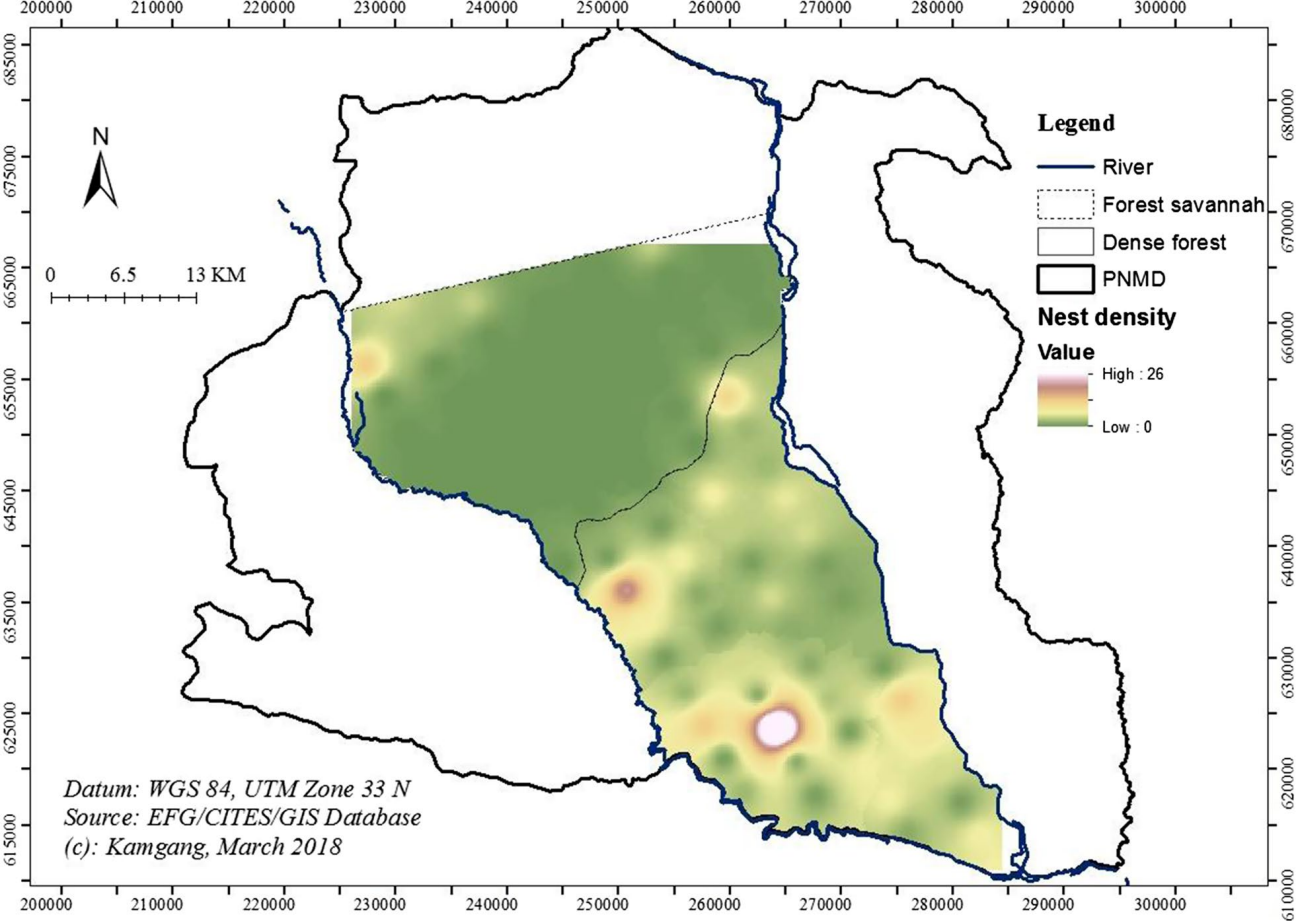
Spatial distribution of chimpanzee nest densities in the core zone of MDNP

The spatial interpolation of the encounter rates of chimpanzee nests is shown in Fig. 7. Chimpanzee nests were found between the Djerem and the Mekié Rivers. Nests were most frequently encountered in the dense forest and especially in the middle, south and north-west of the core zone, and few nests were found in the north.

## Discussion

### Chimpanzee densities

Our findings suggest that gallery forest and colonizing forests are preferred habitats for chimpanzees in the MDNP. The forest-savannah mosaic with associated gallery forests provide suitable habitat for the Nigeria-Cameroon chimpanzee. The nest density estimates varied with strata and the chimpanzee density was similar to the density previously reported in the area, although with a relatively high coefficient of variation. The larger error associated with the chimpanzee density estimate might be explained by the use of a non-site specific nest decay rate as the later depends on the environmental variables of the study site [12] and the intrinsic limitations attributed to the survey method. On 25 of these transects (34% of the total) we found no chimpanzee nests. However, compared to the Ebo Forest in Cameroon where a nest decay rate of 88 days was used to obtain a chimpanzee density estimate of 0.67 animals/km^2^, our density estimate is lower than those from other Nigeria-Cameroon chimpanzee sites (Table 5).

**Table 5:** Population density estimates of the Nigeria-Cameroon chimpanzee (*Pan troglodytes ellioti*) at the Mbam-Djerem National Park compared to other surveys

Site	Decay rate (days)	Chimpanzee/km^2^ (CI)	References
MDNP (dense forest stratum), Cameroon	88	0.88 (0.55–1.41)	This study
MDNP (forest-savannah stratum), Cameroon	120	0.55 (0.19–1.76)	
MDNP (all data combined), Cameroon	221	0.33 (0.12–0.88)	
Ngel Nyaki Forest Reserve, Nigeria	162.8	1.5	[63]
Ngel Nyaki Forest Reserve, Nigeria		1.67	[25]
Taï National Park, Ivory Coast	91.22	0.89	[12]

*CI* 95% confidence interval when available

Although chimpanzee density appears to be low in MDNP, the population may be more stable compared to other sites where hunting is considered to be a major threat [62]. Our density estimate should be considered with caution because specific nest decay rates for MDNP are unavailable. We recommend the MNC method for future surveys because it does not require decay rate and direct observations, even though they are more costly and time-consuming.

### Habitat assessment

We found no evidence that different types of plant species were used for nesting in the dense forest compared with the forest-savannah mosaic, even though considerably more plant species were found in the latter habitat type. *Landolphia* sp. and *Diospyros* sp. were also used by chimpanzees for nesting in Nigeria and Democratic Republic of Congo [23, 63]. The abundance of plant species found in nests in the forest-savannah mosaic might be explained by the high frequency of gallery forests and colonising forests. Both these contain food trees such as *Uapaca guineensis* and are relatively more diverse than dense forest. Thus, chimpanzees may not need to range so far as in dense forest for food, water and nesting materials.

Our results show that most nests occurred in trees, which is consistent with several other field studies of chimpanzee communities in other regions of Africa (i.e. Nigeria [63], Tanzania [16] and Kahuzi Biega National Park [64]). In our study, only 1.20% of nests were on the ground. This was the first time that ground nests have been recorded in the MDNP, and further monitoring is required to understand this behaviour: does it indicate the absence of predators or some aspect of social behaviour? In general, the construction of sleeping nests which are usually more elaborate than the day nests, and ground nesting is rarely observed in unhabituated chimpanzees [65]. Ground nesting has been reported in Senegal in habitats with no or few predators [66] although predators were abundant at the ground nesting site of Bili [23]. In south-east Cameroon, ground nests (3.47% of 1008 nests) were probably the consequence of a lack of nesting trees, or a reaction to hunting with guns or the abundance of terrestrial herbaceous vegetation [67]. It could also indicate that the nest builders were sick [65]. Previous reports of relatively high rates of ground nesting (6.1% of 994 nests) in the Nimba Mountains in Guinea [65] and (3.7% of 37 nests) at Yealé in Ivory Coast [68], have been hypothesized to result either from a male mating strategy, or a regional or seasonal fluctuation in the availability of ground nesting material [68]. Disturbance by humans and seasonal effects may both affect whether chimpanzees construct their nests on the ground or in trees [23]. While Pruetz et al. speculates arboreal nesting as anti-predator adaptation for chimpanzee, there is no evidence that in the absence of predators, chimpanzees switch to ground nesting [64, 69, 70]. Koops et al. suggested that the tendency to build ground nests may be genetically determined in the Nimba Mountains at Seringbara in Guinea. Males may also nest on the ground to guard an oestrous female in a tree above [71].

Over half of the nests were found in gallery forests, highlighting the importance of this habitat type for chimpanzee conservation. At the Ngel Nyaki Forest Reserve in Nigeria, chimpanzees most frequently built nests in gallery forests [72]. Habitat attributes such as elevation also affect nest abundance. Nest encounter rates were higher with increasing elevation between 650 and 800 m a.s.l. in our study, still relatively lower than those of Budongo Forest in Uganda, where they were more likely to be found above 2000 m a.s.l. [21]. Chimpanzee abundance was highly correlated with food availability in the Kibale National Park in Uganda [17] and in the Budongo Forest [21]. In Kahuzi Biega National Park, in Ngel Nyaki Forest Reserve and in Kibale National Park chimpanzees preferred nesting in trees with ripe fruits [64]. Canopy cover was also found to influence the choice of nesting site. Chimpanzees in MDNP appeared to prefer habitat with closed canopy for nesting. Previous studies in Senegal reported that chimpanzees also preferentially chose habitat with closed canopy for nesting [23, 66].

We also found that the nest height was related to the height and dbh of trees (R^2^ = 0.13), as has been described in previous studies [72]. The average dbh of nesting trees at our site was c. 10.30 ± 8.42 cm compared to 54 cm in the Bili-Uele forest in Democratic Republic of Congo [23]. While the average nest height (11.59 ± 7.83 m) was greater compared to 8 m found in Senegal [66] but lower than 20 m found in Nigeria [63]. In the Nigerian study nest height were positively correlated to tree height.

### Chimpanzee distribution

In this study, we found that chimpanzee nests were concentrated in the middle of the core zone and relatively rare in the north and north-east. However, this may vary depending on season and food availability. Using only nests may fail to consider the seasonality of chimpanzees ranging, and should not imply that only areas where nests are observed are valuable for conservation [53, 73]. Further exploration of the effects of human pressure and the density of fruiting trees are required for a better understanding of chimpanzee distribution in the MDNP [17, 74]. Chimpanzees were most abundant in the middle and southern sections of the core zone which are the least accessible to park rangers, and consequently are relatively undisturbed.

The MDNP is the stronghold of Nigeria-Cameroon chimpanzee, noted for its exceptional conservation value [28] and the genetic distinctiveness of its population [10, 75]. Monitoring chimpanzee nesting and feeding sites will continue to be important for efficient conservation planning for this subspecies of great apes. More efforts are therefore needed to assess the chimpanzee nest decay rate to improve the reliability of density estimates. Regular monitoring and patrols should be focused in those areas to sustain this critical chimpanzee population and their habitat into future.

## Conclusions

This study provides the first systematic assessment of the effect of habitat variability on the density of chimpanzees in the MDNP, revealing that gallery forest and colonising forest are preferred by chimpanzees in the core area, while highlighting characteristics of habitat that are positively associated with nest abundance and therefore high conservation importance. Our study indicates that as long as these habitat types are protected, current management practices to maintain savannahs are compatible with chimpanzee conservation. Well-designed surveys are required to assess the sustainability of chimpanzee populations [41, 76]. Currently chimpanzees in the MDNP are becoming increasingly tolerant of humans (Additional file 6). The MDNP offers an excellent opportunity for long-term research on the Nigeria-Cameroon chimpanzee, the least studied great ape subspecies [75] and aimed at understanding and dealing with its potential threats there and elsewhere. To safeguard this area from anthropogenic threats, we recommend that intensive patrols and biomonitoring activities should be focused on the pattern of chimpanzee nesting ecology to prevent threats that could led to almost complete depletion of chimpanzee and other wildlife as has occurred in the Gashaka Gumti National Park and Ngel Nyaki Forest Reserve in Nigeria [63, 77–79]. Further studies need to be focused on nest decay rates and phenology of useful plants (*Berlina* sp., *Diospyros* sp., *Uapaca guineensis*, *Xylopia aethiopica* and *Landolphia* sp.) in order to model chimpanzee abundance and distribution in MDNP. Base on the field observations, human population and chimpanzee both make use of *Xylopia aethiopica* and awareness activities need to be developed to protect this tree species. Our findings were transferred to the park authorities to update the biomonitoring database and measure the progress of chimpanzee conservation in MDNP.

## Additional files

**Additional file 1.** Description of the habitat types. Description was made base on [50] p. 135.

**Additional file 2.** Data used to perfomed density analysis. Data were extracted from the CyberTracker database.

**Additional file 3.** Some plant species found in chimpanzees nests. Plant were identified following a botanist, Garoua wildlife College herbarium and scientific names were checked using (http://www.theplantlist.org/).

**Additional file 4.** Chimpanzee ground nest made with *Marantaceae*. One of our team mate looking for chimpanzee hairs sample on a ground nest. © Missa, 2016.

**Additional file 5.** Data used for the Géneral Linear Model. Data were extracted from the CyberTracker database.

**Additional file 6.** Chimpanzee observed during survey. Chimpanzee (*Pan troglodytes ellioti*) observed during survey in Mbam-Djerem National Park. © Ambahe, 2016.
