# Supplementary material for: The relationship between the abundance of the Nigeria-Cameroon chimpanzee (Pan troglodytes ellioti) and its habitat: a conservation concern in Mbam-Djerem National Park, Cameroon
Source: BMC Ecol. 2018 Oct 1;18:40. doi: 10.1186/s12898-018-0199-3 (PMC6167774; doi:10.1186/s12898-018-0199-3)
Supplement: Supplementary file 1 — Additional file 1. Description of the habitat types. Description was made base on [50] p. 135. [file 12898_2018_199_MOESM1_ESM.docx]

**Habitat type description (White and Edwards 2000), p. 135**

1. *Colonising forest:* expansion of forests through natural succession on savannahs (areas dominated by grasses);
2. *Gallery Forest:* forest running along a river or stream, found both within larger blocks of forest, or isolated in savannah vegetation.
3. *Liana Forest:* forest in which the middle-storey is dominated by lianes, often found close to major rivers;
4. *Marantaceae Forest:* forest with a relatively closed upper canopy, sparse middle storey and dense herb layer on the ground dominated by plants in the Marantaceae and Zingiberaceae ;
5. *Mixed Forest with closed understory:* a forest type which may be similar to old secondary vegetation, with many large trees but dense vegetation cover on the ground (note if under-storey is dominated by lianas, Marantaceae, or other vegetation);
6. *Mixed Forest with open understory:* what many would call 'primary' forest, with many large trees, a high, unbroken canopy and sparse vegetation, cover on the ground, consisting mostly of shrubs
7. *Mixed forest with opened understory or Monodominant forest*: forest with the structure of mixed forest but where one species of tree is noticeably dominant (*Gilbertiodendron,* *Garcinia, Berlinia, Uapaca, Lophira, Aucoumea, Julbernardia*)
