# Supplementary figures and images for: The relationship between the abundance of the Nigeria-Cameroon chimpanzee (Pan troglodytes ellioti) and its habitat: a conservation concern in Mbam-Djerem National Park, Cameroon
Source: BMC Ecol. 2018 Oct 1;18:40. doi: 10.1186/s12898-018-0199-3 (PMC6167774; doi:10.1186/s12898-018-0199-3)

## Slide 1
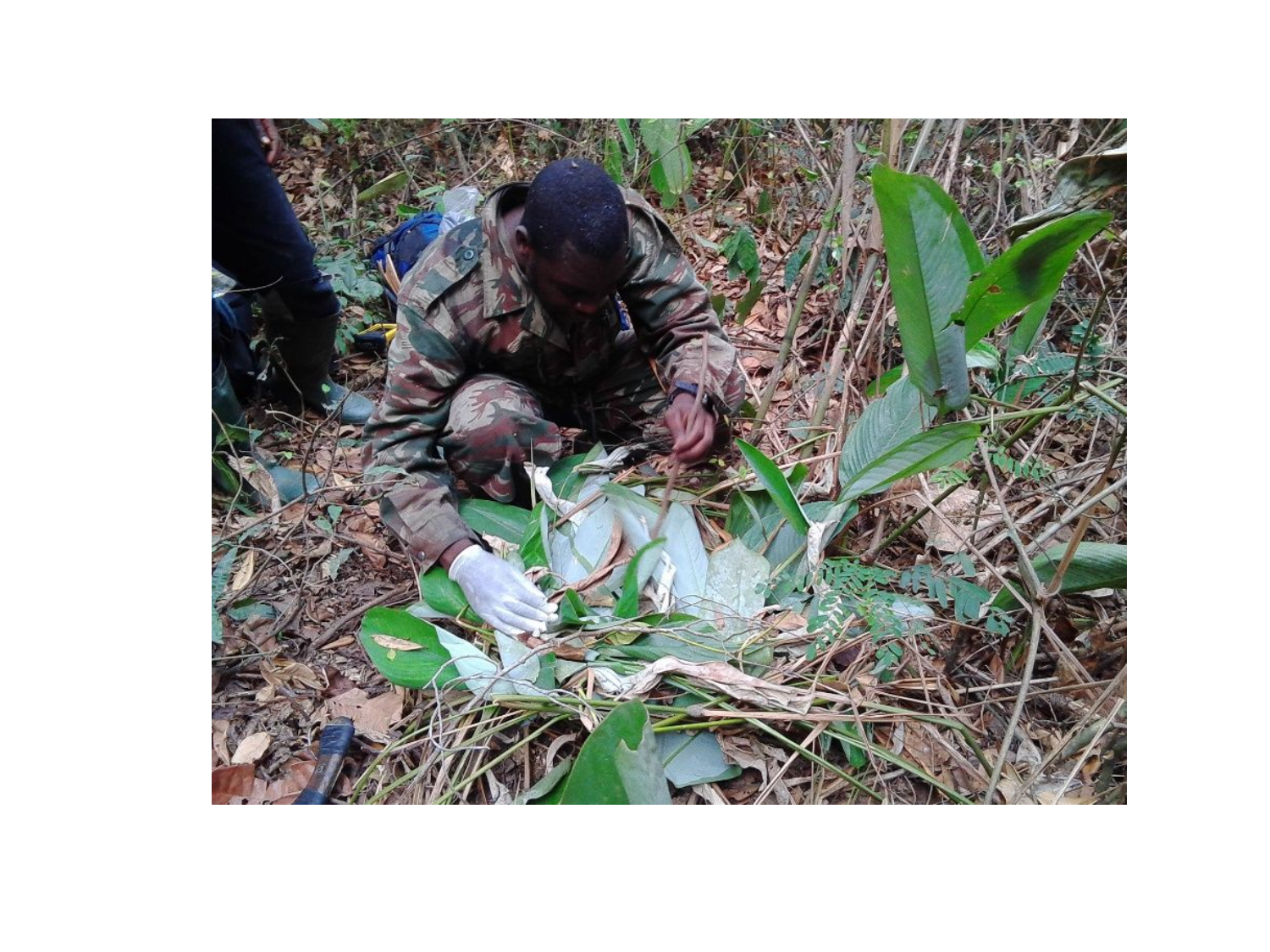

Supplement: Supplementary file 4 — Additional file 4. Chimpanzee ground nest made with Marantaceae. One of our team mate looking for chimpanzee hairs sample on a ground nest. © Missa, 2016. [file 12898_2018_199_MOESM4_ESM.pptx]

## Slide 1
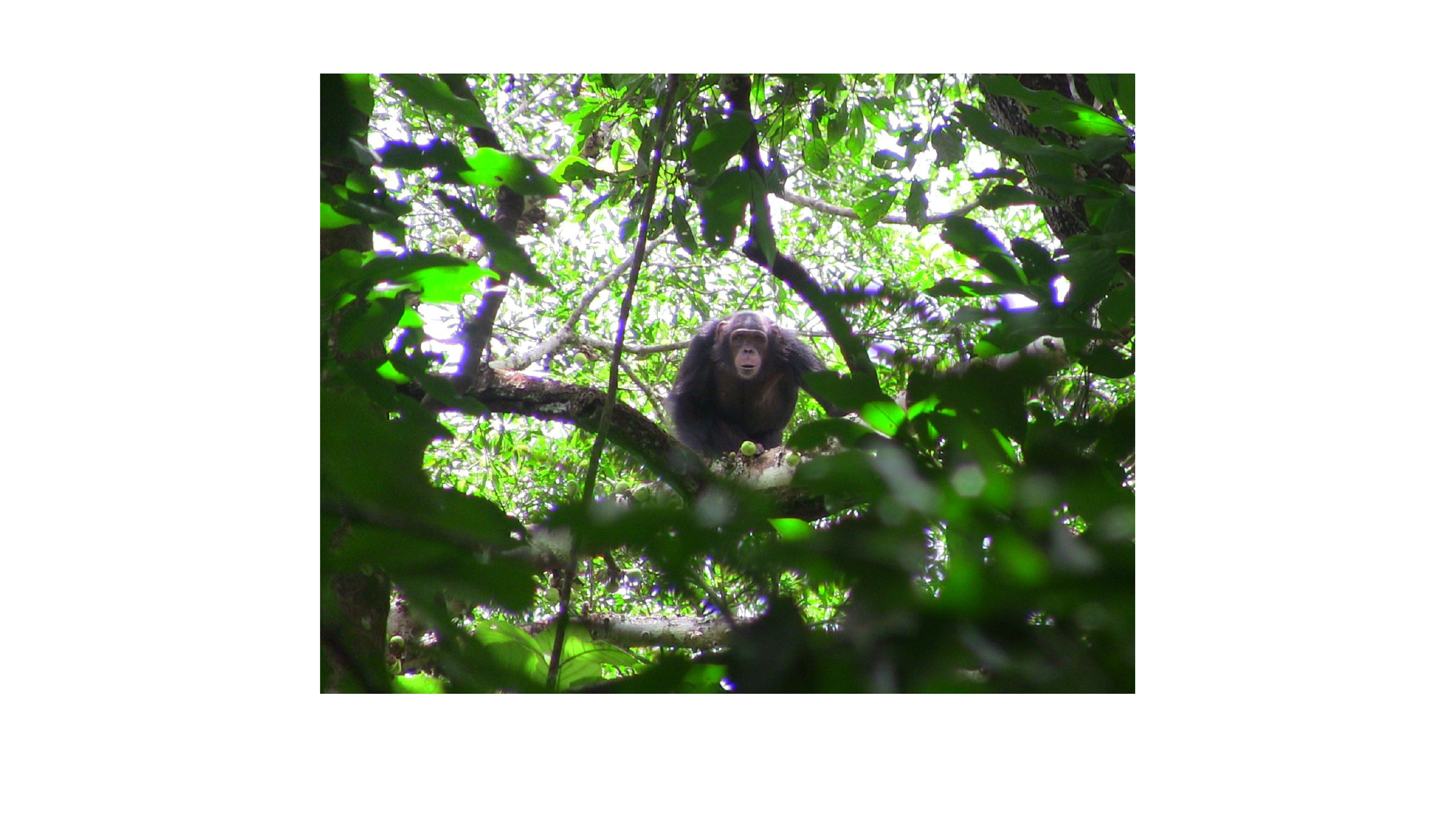

Supplement: Supplementary file 6 — Additional file 6. Chimpanzee observed during survey. Chimpanzee (Pan troglodytes ellioti) observed during survey in Mbam-Djerem National Park. © Ambahe, 2016. [file 12898_2018_199_MOESM6_ESM.pptx]
